# Neoadjuvant Radiation with Concurrent 5-FU Resulting in Complete Pathologic Response in Stage IIIB Squamous Cell Carcinoma of the Urethra

**DOI:** 10.1155/2020/7948538

**Published:** 2020-02-14

**Authors:** Krishna H. Suthar, Meghana Kesireddy, Mark Sides, Amit Correa, Aijan Ukudeyva, Rohit Venkatesan

**Affiliations:** ^1^Department of Internal Medicine, University of Texas Medical Branch, USA; ^2^Department of Internal Medicine, Division of Hematology and Medical Oncology, University of Texas Medical Branch, USA; ^3^Department of Pathology, University of Texas Medical Branch, USA; ^4^Division of Medical Oncology, MD Anderson Cancer Center, USA

## Abstract

Squamous cell carcinoma (SCC) of the urethra is a rare malignancy, comprising less than 1% of all malignancies. The annual age-adjusted incidence of urethral SCC is 4.3 per million in men and 1.5 per million in women. Due to the rarity of the disease, there are a limited number of prospective randomized controlled trials to evaluate the optimal management of locally advanced urethral SCC. Here, we present the case of a 47-year-old man with stage IIIB urethral squamous cell cancer that showed complete clinical and pathologic response to neoadjuvant chemoradiation with only 5-flurouracil after incomplete response to traditional chemotherapy with paclitaxel, ifosfamide, and cisplatin (TIP).

## 1. Case

A 47-year-old African American man presented with a 1-year history of testicular pain and a 6-month history of difficulty urinating. Physical exam revealed a firm mass along the bulbous urethra, which was directly visualized on cystoscopy. Magnetic Resonance Imaging (MRI) of the pelvis showed a well-circumscribed bulbous urethral mass (4.3 × 6.6 × 5.5 cm) that invaded the bilateral corpora cavernosa with prominent bilateral inguinal lymph nodes but no evidence of distant metastasis. He underwent a transurethral biopsy of the mass and was found to have moderately differentiated invasive squamous cell carcinoma involving the corpus spongiosum consistent with Stage IIIB Squamous Cell Carcinoma of the Urethra (cT3, cN1, cM0 per 8^th^ Edition American Joint Committee on Cancer TNM Staging System for urethral cancer). Unfortunately, lymph node biopsy was not obtained at this stage.

He completed three cycles of neoadjuvant therapy with paclitaxel, ifosfamide, and cisplatin (TIP) with the intention of downstaging the tumor [1]. Restaging imaging showed a minimal reduction in the size of the urethral mass to 4.9 × 2.6 cm and stable inguinal lymphadenopathy, confirming incomplete response to initial neoadjuvant chemotherapy.

At this point, the decision was made to offer concurrent chemoradiation with 50 Gy radiation plus 16 Gy boost in conjunction with continuous infusion 5-flurouracil (5-FU) over 8 weeks. Mitomycin was excluded due to the history of neutropenia. Posttherapy imaging demonstrated no discrete urethral mass, only contour irregularity of the cavernosa, and a decrease in the size of inguinal lymph nodes.

**Figure 1:**
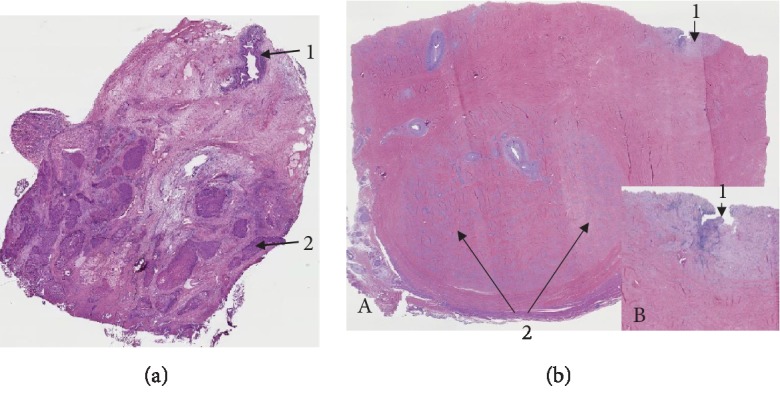
(a) Transurethral resection of tumor before the neoadjuvant chemoradiation therapy (2017). Low magnification shows urethra (1) and moderately differentiated invasive squamous cell carcinoma infiltrating in to the corpus spongiosum (2) (hematoxylin-eosin, original magnification ×4). (b) Status post neoadjuvant chemoradiation (2018). Transverse section of the penile shaft in the area of urethral stenosis. Low magnification shows stenotic urethra with extensive fibrosis and obliteration of corpus spongiosum (1) and extensive fibrosis of corpora cavernosum (2) (A). Note the absence of invasive carcinoma. The inset shows a high power view of the area of urethral stenosis (1) (B). (Hematoxy-lin-eosin, original magnification ×4 (A) and ×8 (B)).

Ultimately, the patient underwent consolidative surgery for curative intent with total penectomy, urethrectomy, cystoprostatectomy with ileal conduit for urinary diversion, and bilateral lymph node dissection 6 months after neoadjuvant chemoradiation due to concerns that concurrent chemoradiation alone would not be enough to achieve a complete response. However, surgical pathology showed complete pathologic response to neoadjuvant chemoradiation with an AJCC Pathologic Stage of pT0, pN0, and M0 (Figure 1).

The patient is currently disease free and remains on active surveillance, with 1-year posttreatment imaging demonstrating no recurrence.

## 2. Discussion

Urethral squamous cell carcinoma (SCC) is a rare malignancy with an annual age-adjusted incidence of urethral SCC which is 4.3 per million in men and 1.5 per million in women [2, 3]. According to the 2018 cancer statistics, malignancies of the urinary system other than the bladder, kidney, and renal pelvis were projected to contribute 3,820 new cases and 960 cancer-related deaths annually [4].

Prospective, randomized controlled trials evaluating optimal management of the locally advanced disease are limited due to the rarity of the disease. Multimodal therapy including chemotherapy, radiotherapy, and surgery is commonly used for invasive urethral SCC, unlike the management of SCC of other organs such as the vulva or anus, which can be managed nonsurgically [5]. Chemotherapy alone or concurrent chemoradiation can be used in the neoadjuvant setting, and cisplatin-containing drug regimens, in particular, have been shown to have good response [5]. Surgical consolidation and wide resection are still commonly used after neoadjuvant therapy [6]. It is important to note that surgery is associated with complaints of reduction in the quality of life, reports of dissatisfaction of genital appearance, need for urinary diversion, and interference with daily activities [7].

There is a clear need to evaluate the potential role of nonsurgical management of urethral SCC. In one such study by Cohen et al., 18 patients with urethral SCC were managed with coordinated chemoradiation as the primary therapy and demonstrated that 83% of patients (15 of 18) had complete response to a primary chemoradiation therapy protocol with 5-year survival rates of 60% and disease-specific survival rates of 83% [8]. The 3 patients who did not respond to primary chemoradiation therapy were managed with salvage surgery. Similar results were seen in a study by Kent et al., with 79% (19 of 26 patients) with complete response to combined chemoradiation with 5-FU, mitomycin, and radiation, and of the responders, 42% had disease recurrence at a median of 12.5 months [9]. While both studies were small in size and did not directly compare outcomes of surgical versus nonsurgical management, it does provide foundations for further studies involving primary chemoradiation therapy.

One must consider the common side effects of chemotherapy and radiation therapy compared to those associated with surgical management. With surgery, the main risks posed to the patient include not only the common surgical risks such as infection and bleeding but also the long-term psychosocial impacts of genitourinary surgery such as body image issues, depression, sexual dysfunction, and need for urinary diversion among others. However, with chemotherapy and radiation therapy, common toxicities such as cytopenia, skin necrosis, diarrhea, neuropathy, and sexual dysfunction can be seen.

The case presented here is unique as the patient did not show radiographic response to a current standard of care regimen, TIP. Based on evidence from the efficacy and safety of chemoradiation in the management of SCC of the head and neck, anus, cervix, and vagina, our patient received neoadjuvant concurrent chemoradiation [9]. Though our patient finally underwent surgery, he had clear evidence of remission on his presurgical imaging, which was later confirmed by pathology. Given the concern for increased morbidity with surgical resection following radiation, there has been limited use of neoadjuvant chemoradiation in urethral SCC, but as our case demonstrates, concurrent chemoradiation without surgery (genital preservation) may suffice as definitive therapy.

This case also raises the implication that single-agent chemoradiation may be equivalent in efficacy as double-agent therapy in the potential management of urethral cancer as our patient responded to single-agent 5-FU with radiation. There have been cases reported of bulky urethral tumors that had a complete response to chemoradiation therapy using combination chemotherapy with 5-flurouracil and cisplatin [10].

To our knowledge, this is a unique case of radiographic and confirmed complete pathologic response with chemoradiotherapy with 5-FU alone for urethral SCC following the lack of response to standard neoadjuvant chemotherapy with TIP. While our patient ultimately had surgery per current standard of care, the pathology on the surgical specimens demonstrated no evidence of disease, raising the question if surgical resection was required given the notable response after chemoradiation seen on imaging. Thus, there may be a role for nonsurgical management of locally advanced SCC urethral, which needs to be further studied.
